# Comprehensive Study in the Inhibitory Effect of Berberine on Gene Transcription, Including TATA Box

**DOI:** 10.1371/journal.pone.0023495

**Published:** 2011-08-22

**Authors:** Yugang Wang, Michael M. Kheir, Yushuang Chai, Jun Hu, Dongming Xing, Fan Lei, Lijun Du

**Affiliations:** 1 Protein Science Laboratory of the Ministry of Education, Laboratory of Pharmaceutical Sciences, School of Life Sciences and School of Medicine, Tsinghua University, Beijing, China; 2 Department of Chemistry, University of Michigan, Ann Arbor, Michigan, United States of America; University of South Florida College of Medicine, United States of America

## Abstract

Berberine (BBR) is an established natural DNA intercalator with numerous pharmacological functions. However, currently there are neither detailed reports concerning the distribution of this alkaloid in living cells nor reports concerning the relationship between BBR's association with DNA and the function of DNA. Here we report that the distribution of BBR within the nucleus can be observed 30 minutes after drug administration, and that the content of berberine in the nucleus peaks at around 4 µmol, which is twelve hours after drug administration. The spatial conformation of DNA and chromatin was altered immediately after their association with BBR. Moreover, this association can effectively suppress the transcription of DNA in living cell systems and cell-free systems. Electrophoretic mobility shift assays (EMSA) demonstrated further that BBR can inhibit the association between the TATA binding protein (TBP) and the TATA box in the promoter, and this finding was also attained in living cells by chromatin immunoprecipitation (ChIP). Based on results from this study, we hypothesize that berberine can suppress the transcription of DNA in living cell systems, especially suppressing the association between TBP and the TATA box by binding with DNA and, thus, inhibiting TATA box-dependent gene expression in a non-specific way. This novel study has significantly expanded the sphere of knowledge concerning berberine's pharmacological effects, beginning at its paramount initial interaction with the TATA box.

## Introduction

Berberine (BBR) is a natural quaternary ammonium salt from the group of isoquinoline alkaloids. Modern pharmacological research have discovered that BBR is a multi-pharmacological molecule exhibiting anti-inflammatory [1], cell cycle inhibition [2], anti-diabetic [3] and neuroprotective properties [4]. According to several medical studies, BBR has activities against and has already been used to treat dysentery, hypertension, inflammation, and liver disease in clinics [5], [6].

Since the 1960s, many researchers have been interested in the interaction between BBR and cell nuclei [7]-[10]. These previous studies have established that BBR belongs to a class of alkaloid DNA intercalators and is cell-permeable so that it can be transported into the cell via a cation transporter and expelled by P-glycoprotein [11], [12]. However, no prior study has revealed to us the complete account describing what occurs once BBR has been transported into a living cell. What is the relationship between BBR's DNA intercalating character and its broad range of activities?

In this research, a complex and dynamic process of BBR entering from a medium into a living cell nucleus was described for the first time. The transcriptional levels of the global genome and several artificial plasmids, including TATA box-dependent and TATA box-independent genes, were compared before and after BBR addition. Coupled with data from a cell-free transcription system, the disruptive effect observed via EMSA and ChIP of BBR binding to the TATA box on the association between TBP and the TATA box allows us to hypothesize that BBR can suppress the transcriptional ability of genes in a non-specific way when it distributes throughout a living cell nucleus. One of the underlying mechanisms is the stereospecific blockade and the spatial conformational change in DNA and chromatin caused by the association between BBR and DNA, which blocks TBP from binding to the TATA box, ultimately suppressing the initiation of gene transcription. The TATA box is, therefore, one of BBR's crucial targets for suppression of DNA transcription in living cells.

## Materials and Methods

### Preparation of Chromatin & Genomic DNA from Rat Liver

Male albino Sprague-Dawley rats weighing around 220±10 g and about 2 months old in age were used throughout this study. Nuclei were isolated from the homogenized liver by using a method described previously by Blobel and Potter [13]. The quality of the collected nuclei was tested according to the protocol described by Bonner [14]. Genomic DNA was obtained from the homogenized liver by using a TIANamp Genomic DNA Kit (Tiangen Biotech, China). All the biological experiments were conducted under the supervision and approval of Institutional Animal Care & Use Committee of Tsinghua University and Animal Welfare & Ethics Committee of Tsinghua University (Approval ID: 2010-DuLJ-Neuron).

### Cytotoxicity assay

The PC12 cells (obtained from the tumor cells library of Chinese Academy of Medical Sciences commercially) used in this study derive from a cell line from a pheochromocytoma of the rat adrenal medulla. The cytotoxicity of BBR in PC12 cells was evaluated by an MTT (3-(4,5-dimethylthiazol-2-yl)-2,5-diphenyltetrazolium bromide) assay and a Lactate dehydrogenase (LDH) cytotoxicity assay. The MTT assay was performed according to the method described by Mosmann [15] and the LDH cytotoxicity assay was performed according to the manufacturer's guidelines (CytoTox 96 Non-Radioactive Cytotoxicity Assay, Promega, USA).

### Plasmid Transfection

Plasmids driven by the TPH2 promoter and IgG promoter were constructed previously [16], [17] and stored at the Laboratory of Pharmaceutical Sciences at Tsinghua University. Plasmids driven by the PPARγpromoter were kindly provided by Dr. Fan Lei. pEGFP-N1 and pDsRed-Express-N1 were kindly provided by Professor. Yeguang Chen. pEGFP-N1-hTBP was constructed by fusing the human TATA binding protein with green fluorescent protein. Plasmids were transfected into PC12 cells by a Lipofect Transfection Reagent (Tiangen Biotech, China) according to the manufacturer's guidelines.

### Quantitative analysis of RNA

The total RNA from PC12 cells was extracted using RNAprep pure Cell/Bacteria Kit (Tiangen Biotech, China). The total content of RNA was measured by using a GeneQuant 100 Spectrophotometer (General Electric Company, Germany).

### Real-Time polymerase chain reaction

Real-Time PCR was conducted using a RealMasterMix (SYBR Green) Kit (Tiangen Biotech, China). Primers for detecting GFP were: forward 5′-GCAGAAGAACGGCATCAAGG-3′ and reverse 5′- CGGACTGGGTGCTCAGGTAG-3′; primers for detecting RFP were: forward 5′- AGAAGAAGACTATGGGCTGG-3′ and reverse 5′- CGTTGTGGGAGGTGATGT-3′; primers for detecting the TATA box-containing sequence from the CMV promoter were: forward 5′- CTCACGGGGATTTCCAAGTC -3′ and reverse 5′- CTGACGGTTCACTAAACCAGCT -3′. Signals from SYBR Green were recorded and analyzed by the CFX96 Real-Time PCR System (Bio-Rad, USA).

### Absorbance and Fluorescence Measurement

The analysis of the interaction between BBR and testing components (plasmid DNA, genomic DNA, and chromatin) was performed via fluorometric titration on a RF-5301PC Spectrofluorometer (Shimadzu, Japan). A 3.0 mL solution, containing 10 µmol of BBR in 10 µmol of Tris-HCl (pH 8.0), was titrated by successive additions of solution composed of testing components. Titrations were done manually by adding the corresponding storage solution. The fluorescence emission spectra was recorded at a temperature of 20°C in the wavelength range of 400–650 nm and with an exciting wavelength of 456 nm. To test the interaction of BBR with proteins that comprise the chromatin, 3.1 µmol of chromatin were titrated with discontinuous additions of BBR solution. The fluorescence emission spectra were recorded in the wavelength range of 280–400 nm with the exciting wavelength at 280 nm. All other experimental conditions were identical to those stated above. Appropriate blanks corresponding to the buffer solution were subtracted to correct for background fluorescence.

### Confocal microscopy

PC12 cells were used in the experiment when they reached 70% confluence. BBR-treated (2.69 µmol) cells were then fixed. Nucleus staining of fixed cells was done with propidium iodide (PI) and acridine orange (AO). For recording the dynamic process of BBR entering the nucleus of living cells, the “stack” and “time series” modes was used with a time interval of 1.2 min, and neither fixation nor nucleus staining proceeded. Images were taken with a Zeiss LSM 710 Confocal Microscope (Carl Zeiss, Germany) and analyzed by Zen Light Edition Software.

### Isothermal Titration Calorimetry

Isothermal calorimetric measurements were performed in a NANO-ITC 5300 (TA Instruments, USA) at 20°C, equipped with a temperature controller. Titration of BBR against different testing components was performed by injecting BBR (1000 µmol) into the testing components. Twenty-five titrations were performed with a volume of 10 µL each time. A blank experiment in which BBR was injected into buffer (10 mmol Tris-HCl, pH 8.0) with no testing component was performed to correct for dilution in the data. The background was subtracted from the measured heat data and the corrected heat data were plotted against the molar ratio as well as analyzed using the manufacturer's software, yielding the stoichiometry n (number of berberine molecules/number of base pairs), the equilibrium dissociation constant (K_d_ = 1/K_a_), and the enthalpy (_Δ_H).

### Dynamic Light Scattering

Dynamic light scattering experiments were performed using a DynaPro Dynamic Light Scattering Instrument (Wyatt Technology Corporation, USA), equipped with a laser of wavelength 832.6 nm. The size of the particles was calculated using the manufacturer's software.

### Circular Dichroic Spectroscopy

Circular dichroic (CD) measurements were made in a JASCO J-500 Spectropolarimeter (Jasco Corporation, Japan) at 20°C, equipped with a temperature controller. The CD scans were recorded within the wavelength range of 220–350 nm, at a sensitivity set to 10 mdeg, with a scan speed of 20 nm per minute, and with a step size of 0.5 nm. The time constant was 1 s and bandwidth was 0.2 nm. All measurements were made in a cuvette of 1 mm path length in a reaction volume of 200 µL in 10 mmol Tris-HCl (pH 8.0) at 20°C. All spectra depict the average of three runs. They were smoothed within permissible limits by the instrument's built-in software.

### Analysis of GFP expression by flow cytometry

A BD Calibur Flow Cytometer system (BD Inc, USA) was used to analyze the expression of GFP.

### Quantitative analysis of berberine in a living cell by high performance liquid chromatography (HPLC)

The HPLC system and procedure used for quantitative analysis of the BBR content in living cells were identical to those described in previous literature [18]. The concentration of BBR in a living cell was calculated by using the following equation: 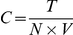, where C is the concentration of BBR in a living cell, T is the total content of BBR detected in the cell population, N is the number of cells detected by the hematocyte counter, and V is the volume of a living cell, which was assumed to be 1.5×10^−12^/L [19]. The concentration of BBR in the nucleus was computed according to the following equation: 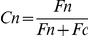, where Cn is the concentration of BBR in the nucleus, Fn is the fluorescent intensity of BBR in the nucleus, and Fc is the fluorescent intensity of BBR in the cytoplasm.

### Transcription analysis in a cell-free system

The effect of BBR on the transcription of DNA was investigated using the HelaScribe Nuclear Extract in vitro Transcription System (Promega, USA). The protocol used in this experiment was modified according to the manufacturer's guidelines: the positive control DNA was used as a template in all groups and the volume used was 2 µL; RNA product was washed twice with 70% ethanol to flush the radioactive rGTP which is not incorporated in the transcription product; the content of RNA product was measured using a MicroBeta Jet-Microplate Scintillation and Luminescence Counter (PerkinElmer, USA).

### Electrophoretic mobility shift assay

Forty-eight hours after the plasmid pEGFP-N1-hTBP transfected into PC12 cells, nuclear extracts were prepared by using a NE-PER Nuclear and Cytoplasmic Extraction Reagent Kit (Pierce, USA) according to the manufacturer's guidelines. The TATA box-containing oligonucleotide was synthesized as complementary oligodeoxyribonucleotide strands and labeled by a Biotin 3′ End Labeling Kit (Thermo, USA). The TATA box sequence has been described previously [20]: 5′-GAAGGGGGGCTATAAAAGGGGGTG-3′.

The association between the TATA binding protein (TBP) and the TATA box was assessed using the LightShift Chemiluminescent EMSA Kit (Pierce, USA). The labeled TATA box probe was incubated with different dosages of BBR just before the binding reaction. The binding reaction and gel detection were performed according to the manufacturer's guidelines.

### Chromatin immunoprecipitation and Western blotting

Forty-eight hours after the plasmid pEGFP-N1-hTBP transfected into PC12 cells, the cells were grouped and sampled. The content of TATA box-containing sequences in the CMV promoter that were associated with TBP was detected using an EZ-ChIP Chromatin Immunoprecipitation Kit (Millipore, US) according to the manufacturer's guidelines. The protocol for Western blotting was identical to one described previously [21]. The antibody used in ChIP and Western blotting was ChIP grade (ab51841) from Abcam (Abcam, USA).

### Statistical analysis

All values were expressed as mean ± S.D. Data were statistically analyzed using one-way ANOVA followed by Dunnet's test. P<0.05 was accepted as statistical significance.

## Results

### Berberine can enter the cell nucleus within 30 minutes in a dynamic process

**Figure 1:**
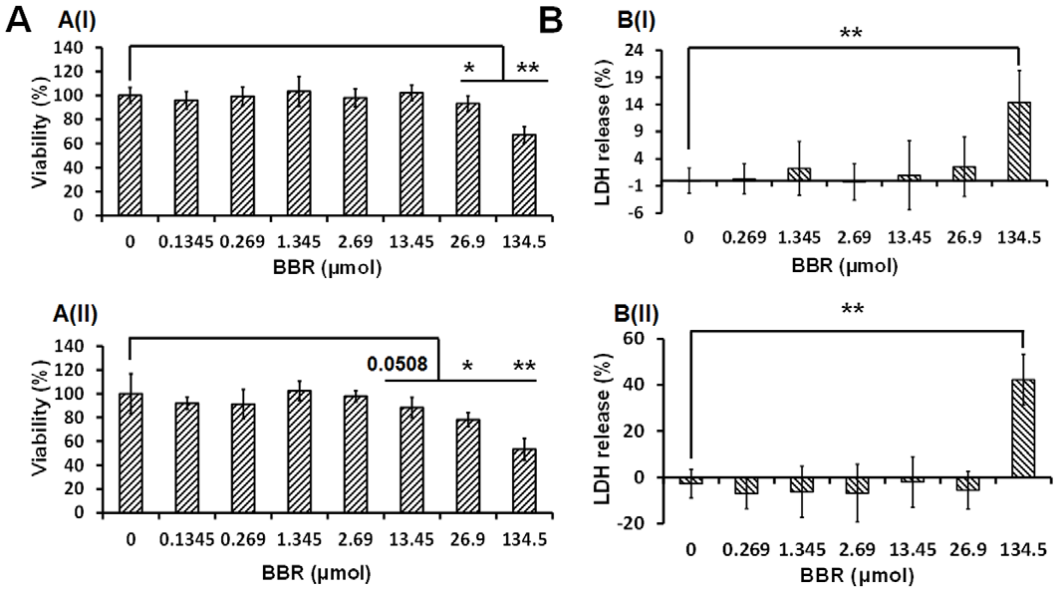
Cytotoxicity assay of berberine. (A) represents the cytotoxicity of BBR from the MTT assay: A(I) and A(II) represent the cytotoxicity of BBR to PC12 cells 12 hours and 24 hours after drug administration, respectively; (B) represents the LDH release of PC12 cells after BBR addition: B(I) and B(II) represent the amount of LDH released from PC12 cells 12 hours and 24 hours since BBR incubation. In the MTT assay, the group with 0 µmol of BBR was considered the control group; in LDH release assay, the spontaneous release of LDH from the BBR-free group (0 µmol) was considered the control group. The percentage of LDH release was calculated by the equation: LDH release (%) = (Experimental LDH release-Spontaneous LDH release)/Maximum LDH release. * p<0.05, ** p<0.01 vs control. Data were presented as mean ± S.D. from twelve independent experiments. (n = 12).

**Figure 2:**
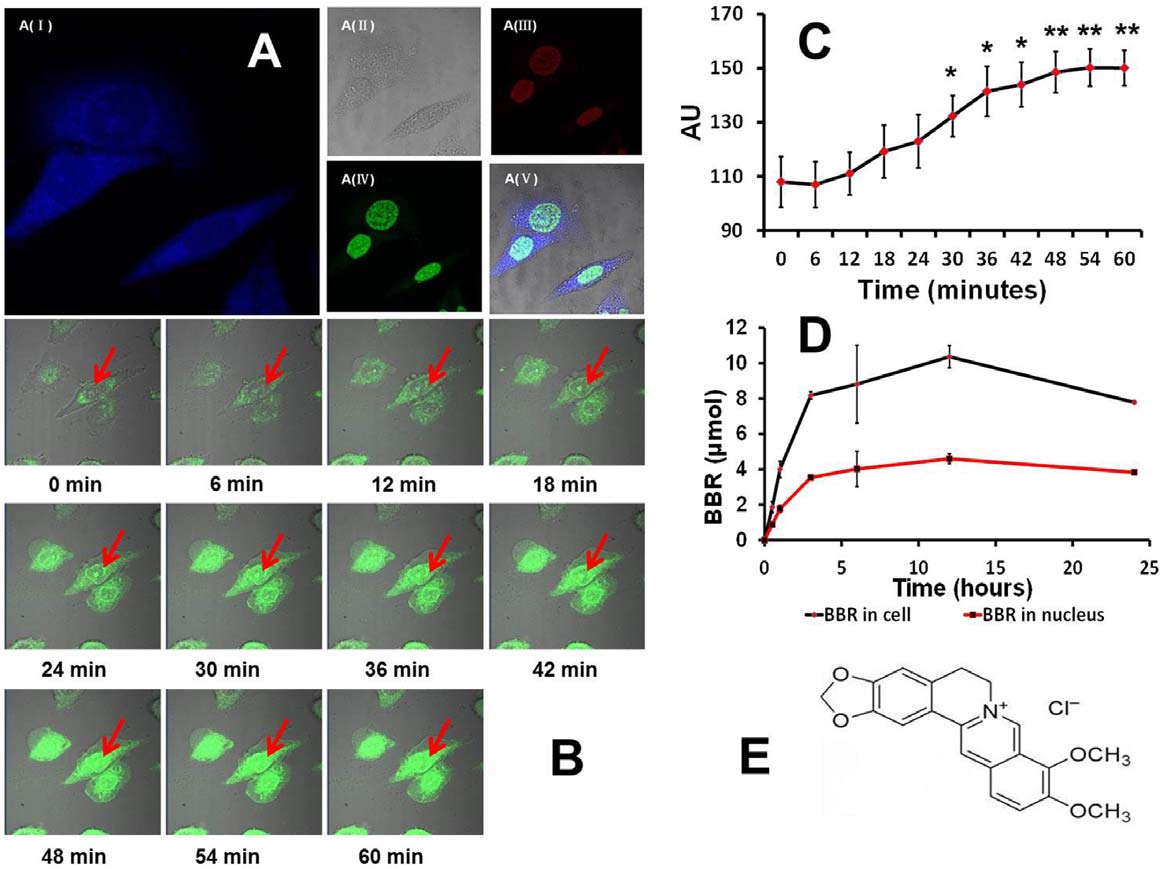
Distribution of berberine in a living cell. (A) (from I to V) depicts images of the subcellular location of BBR in PC12 cells one hour after BBR administration. The fluorescence of BBR is shown in blue in A (I); A (II) represents PC12 cells in visible light; A (III) and A (IV) represents the nucleus stained by PI and AO, respectively; figure A (V) is the merged image of A (I), A (II), A (III), and A (IV). (B) represents the process of BBR entering the nucleus. The fluorescence of BBR is shown in green in this figure, and the red arrow points to the nuclear region. All of the images in (B) were taken under the same conditions with a time interval of 1.2 min. (C) represents the fluorescence intensity of BBR in the nucleus. * p<0.05, ** p<0.01 vs control (0 minutes after BBR administration). (D) represents the time-resolution change of berberine in live cells and the nucleus, which was detected by HPLC. (E) represents the chemical structure of BBR. Data were presented as mean ± S.D. from three independent experiments (n = 3).

To determine whether BBR could enter cells, we observed the distribution of BBR in living cells by a confocal assay. A safe dose of berberine (2.69 µmol) was used throughout the entire study based on the results of a cytotoxicity assay (Fig. 1 A (I) & A (II); B (I) & B (II)). The confocal microscope depicted detailed images regarding BBR distribution in living cells (Fig. 2 A (I)). Continuous observation revealed that BBR can enter the nucleus in a dynamic process within 30 minutes after drug administration (Fig. 2 B and C) and the highest peak of BBR content in the nucleus, at around 4 µmol, was observed by an HPLC assay approximately 12 hours after the drug was added into the culture medium (Fig. 2 D).

### Berberine can interact with genetic components after entering the nucleus

**Figure 3:**
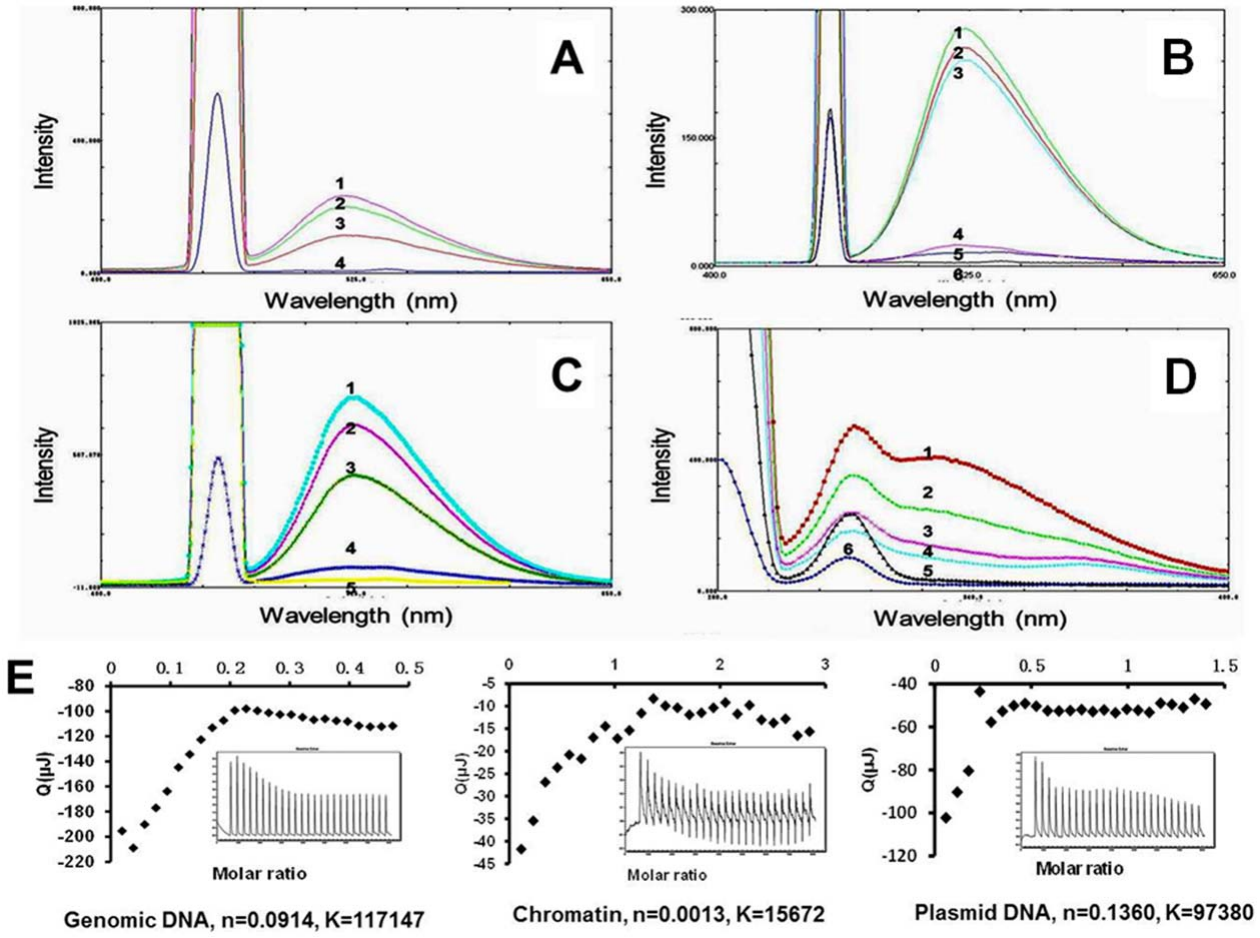
Interaction between berberine and genetic components. (A) represents the fluorescence spectrum of BBR (10 µmol) associating with chromatin (15.3 µmol, spectrum 3; 30.6 µmol, spectrum 2; 45.9 µmol, spectrum 1; 0 µmol, spectrum 4); (B) represents the fluorescence spectrum of BBR (10 µmol) associating with plasmid (138.2 µmol, spectrum 1; 92.1 µmol, spectrum 2; 44.6 µmol, spectrum 3; 0 µmol, spectrum 4; spectrum 5 and spectrum 6 represent the fluorescent spectrum of plasmid and buffer, respectively); (C) represents the fluorescence spectrum of BBR (10 µmol) associating with genome (30.8 µmol, spectrum 3; 61.6 µmol, spectrum 2; 92.4 µmol, spectrum 1; 0 µmol, spectrum 4; spectrum 5 represents the fluorescence spectrum of buffer); (D) represents the fluorescence spectrum of tryptophan in chromatin (3.1 µmol) associating with BBR (0 µmol, spectrum 1; 10 µmol, spectrum 2; 20 µmol, spectrum 3; 30 µmol, spectrum 4; spectrum 5 and spectrum 6 represent the fluorescence spectrum of BBR and buffer, respectively). According to (A), (B), and (C), the exciting wavelength is 456 nm and the emission wavelength is 548 nm. The exciting and emission wavelength in (D) is 280 nm and 330 nm, respectively. (E) portrays the isothermal calorimetric measurements revealing the detailed parameters concerning the association of BBR with genomic DNA, chromatin, and plasmid DNA, respectively.

The nucleus is the domain that embodies genetic components and is the prime location where these components exert their functions. Although previous research have investigated the interaction of BBR (Fig. 2 E), a DNA intercalator, with nuclei and artificial DNA models [9], [10], [22], no data systematically describing the interaction of BBR with native genetic components directly extracted from living cells have been made available despite vast interest in this subject. This association of BBR with testing components was first probed by fluorescence spectroscopy. An increase in quantum yield in the fluorescence spectrum of BBR is the preliminary evidence for the binding of BBR to different testing components (Fig. 3 A, B and C), which is in accordance with a previous report concerning the association of BBR with artificial DNA models [23]. Furthermore, the fluorescence quenching of tryptophan indicated that BBR not only interacts with the DNA in chromatin but also associates with the proteins contained in chromatin (Fig. 3 D).

For additional detailed information regarding the association between BBR and testing components, isothermal calorimetric titrations were performed to determine the binding parameters and associated energetics. Association constants and binding stoichiometry values between BBR and testing components obtained from ITC measurements are shown in figure 3 E. From the binding stoichiometry, the number of base pairs associated with one BBR molecule (including the genome and plasmids) was almost 100-fold greater than in chromatin, which is in accordance with the number of base pairs (146 bp) packed into one nucleosome, suggesting that BBR could associate with DNA and proteins in chromatin simultaneously but with different affinities. Moreover, the packaging arrangement of nucleosomes in chromatin would affect BBR's accessibility to the portion of DNA packaged in the interior of the nucleosome.

### Berberine can induce spatial conformational changes in chromatin and DNA

**Figure 4:**
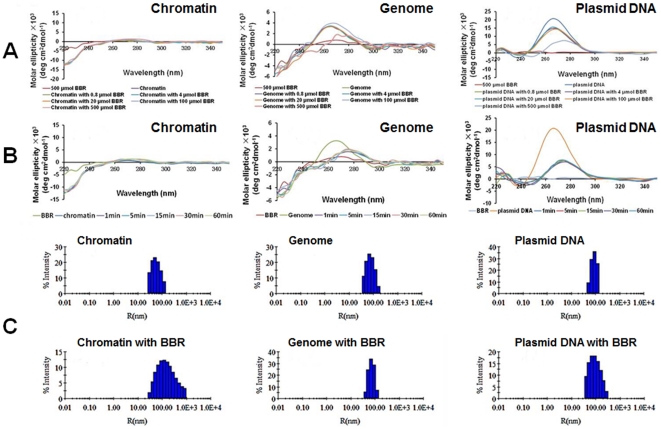
Spatial conformational change induced by berberine. (A) represents the dose-dependent alterations in circular dichroic spectrum of genome, chromatin, and plasmid, respectively. (B) represents the time-dependent alterations in circular dichroic spectrum of genome, chromatin, and plasmid, respectively. (C) represents the effect of BBR (500 µmol) on the intensity distribution (%) of chromatin (92.4 µmol), genome (423.2 µmol), and plasmid (808 µmol) in size (diameter) obtained from DLS measurements, respectively.

According to the CD spectroscopy of BBR interacting with native chromatin, genome, and plasmid DNA, BBR influenced the CD spectra of DNA (genomic DNA and plasmids) in a dose-dependent manner (Fig. 4 A) and the process of this modification was completed in one minute (Fig. 4 B). However, no alterations were observed in the CD spectra of chromatin after BBR administration. Dynamic light scattering measurements were carried out to further characterize the ultra-structural consequences of BBR's association with chromatin, genome, and plasmid DNA. There was no significant change in the mean hydrodynamic diameter of genomic DNA and plasmid DNA in the presence of BBR (Fig. 4 C). However, an aggregation of chromatin was observed, suggesting that the intercalation of this alkaloid into DNA's minor groove [22] has no impact on the tertiary structure of DNA; rather, the interaction between BBR and proteins associated with chromatin could directly induce the alteration in the chromatin's diameter.

### Berberine can inhibit plasmid expression in cells

Given that berberine can induce spatial conformational changes in DNA (genome and plasmids) by direct association as well as induce the diameter aggregation of chromatin by its association with proteins in chromatin, what ramifications would result from these associations?

**Figure 5:**
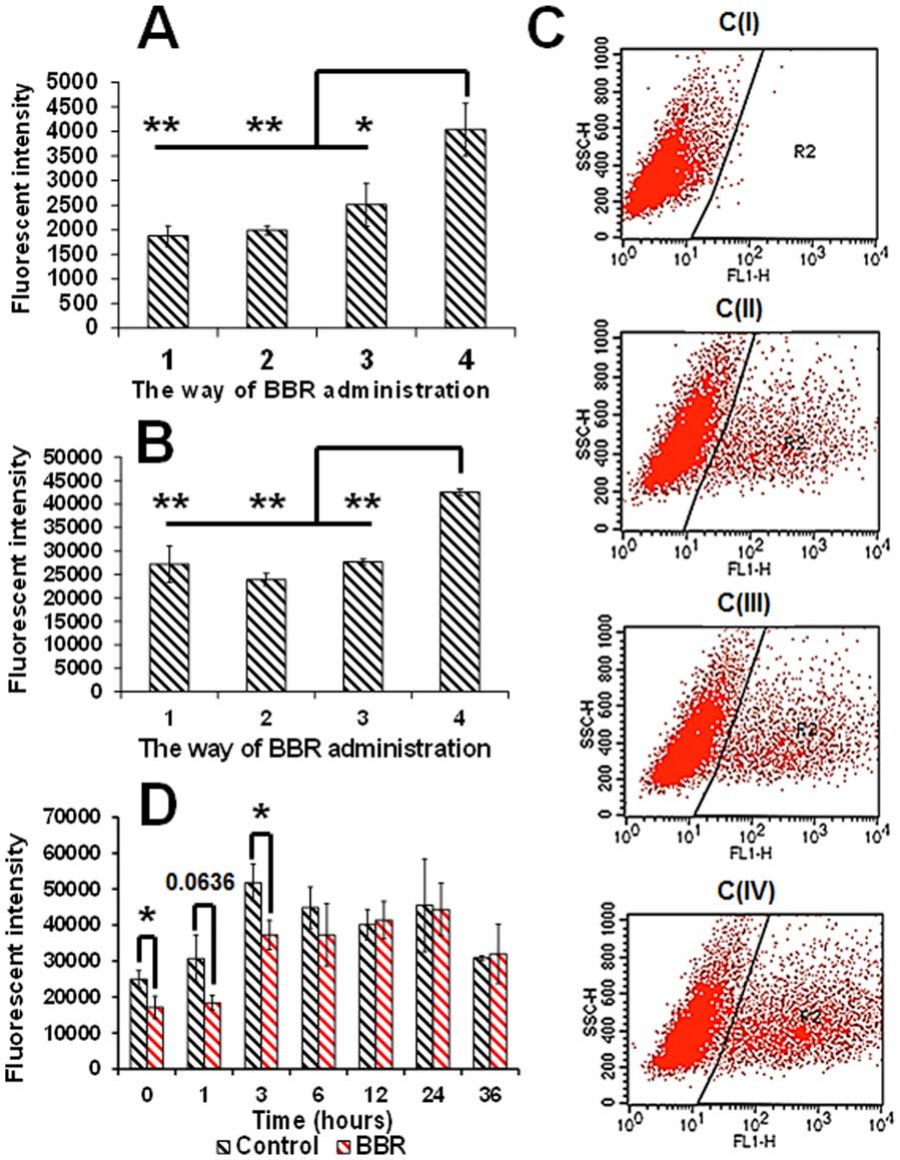
Suppressive effect of BBR on the expression of green fluorescent protein in PC12 cells. (A) and (B) represent the suppressive effect of BBR on the expression of GFP 12 hours and 24 hours after drug administration, respectively. The value on the x-axis represents the method of drug administration: (1) denotes that BBR has been incubated with PC12 for 1 hour before transfection; (2) denotes that BBR has been incubated with plasmid for 5 minutes before transfection; (3) denotes that BBR was administered 6 hours after transfection; (4) denotes transfection without BBR administration. (C) portrays the method of data collection according to the flow cytometry experiments. C (I), C (II), C (III), and C (IV) represent the method of data collection of PC12 cells without GFP expression, GFP expressing PC12 cells with BBR treatment for 12 hours, GFP expressing PC12 cells with BBR treatment for 24 hours, and GFP expressing PC12 cells without BBR treatment, respectively. The R2 region in the images in (C) characterizes the definition of GFP expressing cells in the cell population. (D) depicts the recovery of GFP expression after the elimination of 12 hour-BBR-treatment (n = 3). (Compared with group 4, * p<0.05, ** p<0.01). Fluorescence intensity =  G×M, where G indicates the number of fluorescent cells and M is the mean fluorescence intensity of fluorescent cells.

Storing and expressing genetic information are fundamental functions of DNA in the nucleus. In order to explore the consequences of the spatial conformational alterations in DNA induced by BBR, plasmids were incubated with BBR during plasmid transfection. By employing flow cytometry, data from different groups revealed that BBR could effectively suppress the expression of the plasmid in living cells at both 12 hours and 24 hours after plasmid transfection, irrelevant of the method of drug administration (Fig. 5 A, B and C). This suppressive effect can be eliminated when BBR-treatment is removed (Fig. 5 D).

### Berberine can suppress DNA transcription in a living cell system

**Figure 6:**
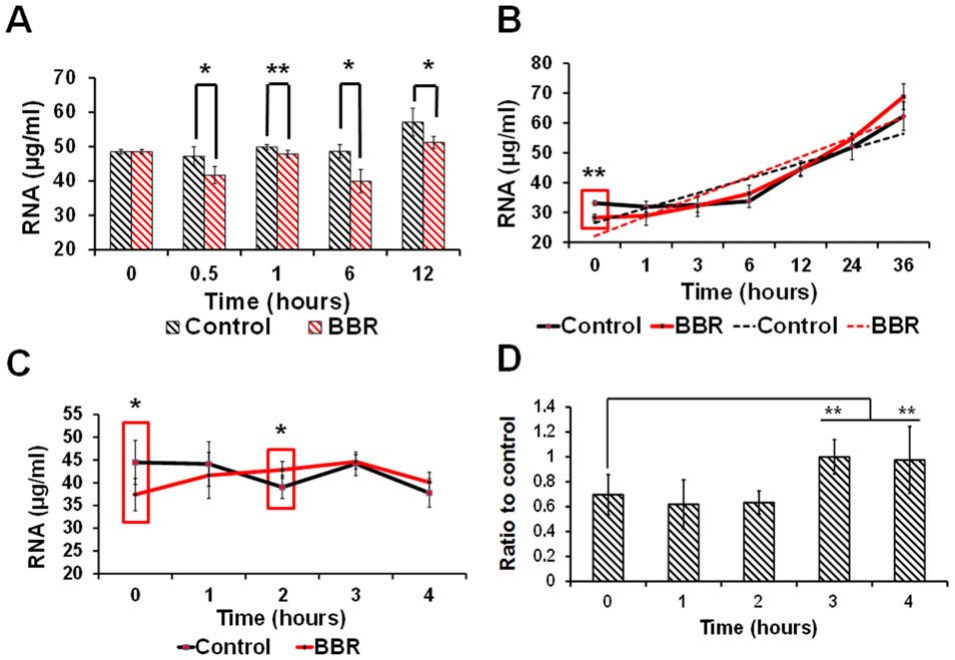
The suppressive effect of berberine on gene transcription in live cells. (A) portrays the time-dependent effect of BBR on gene transcription on a global level. (B) shows the time-dependent recovery of the global RNA level after the elimination of a 12 hour-BBR treatment. The linear equation of the control group in (B) is: *y* = 4.9836×*+21.516*, R^2^ = 0.8162; the linear equation of the BBR group in (B)is: *y* = 6.6219×*+15.472*, R^2^ = 0.8941. (C) displays the protective effect of BBR on the global RNA level. (D) depicts the protective effect of BBR on the mRNA level of the transfected artificial plasmid, pEGFP-N1. The dosage of BBR in all of the experiments was 2.69 µmol. Data in D was the ratio of target gene in the BBR group to the same target gene in the BBR-free group at the same time-point (control group). * p<0.05, ** p<0.01 vs the corresponding control indicated above. Data were presented as mean ± S.D. from four independent experiments (n = 4).

According to the central dogma, transcription of DNA is the first step of protein-encoding gene expression, followed by the translation of mature mRNA into protein. In order to obtain more detailed information regarding the effect of BBR on gene expression, the content of RNA in BBR-treated groups was compared to that in the non-BBR-treated group. Quantitative results of their corresponding global RNA levels revealed that BBR could significantly suppress gene transcription on a global scale (Fig. 6 A) and that this inhibitory effect could be reversed with the elimination of BBR (Fig. 6 B).

Two coupled yet distinct dynamic processes determine the RNA content in living cells: RNA synthesis and RNA degradation. In order to clearly decipher whether the decreased content of RNA in the BBR-treated groups was due to an inhibition of RNA synthesis or due to an accelerated degradation induced by BBR, the half-life of RNA in living cells was examined after transcriptional inhibition. Data from this experiment indicated that BBR could effectively protect the mRNA of plasmid DNA from degradation in living cells (Fig. 6 C). Furthermore, this protective effect can be observed on a global scale (Fig. 6 D) where the content of global RNA in the BBR-treated groups was higher than the global RNA level in the control group two hours after transcription inhibition. Therefore, it is confirmed that BBR does indeed decrease the content of RNA by suppressing gene transcription.

**Figure 7:**
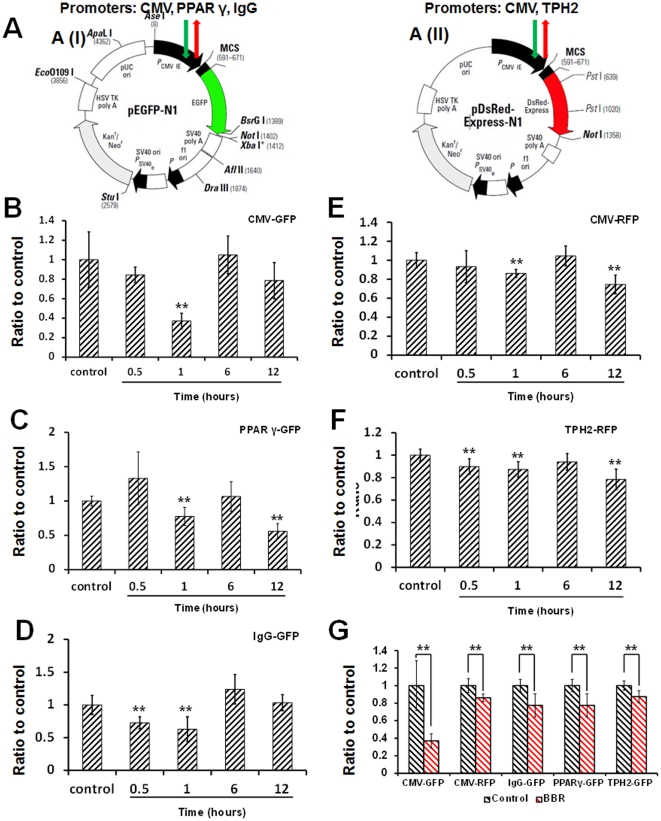
The suppressive effect of BBR on the transcription of artificial plasmids. A (I) and A (II) signify the type of artificial plasmid model used in this study. (B), (C), (D), (E), and (F) represent the effect of BBR on CMV-GFP, PPARγ-GFP, IgG-GFP, CMV-RFP, and TPH2-RFP plasmids, respectively. (G) illustrates the suppressive effect of BBR on the expression of these five plasmids 1 hour after drug administration. Data is the ratio of the target gene in the BBR group to the same target gene in the BBR-free group at the same time-point (control group) * p<0.05, ** p<0.01 vs the corresponding control indicated above. Data were presented as mean ± S.D, from three independent experiments (n = 3).

To further validate the suppressive effect of BBR on gene transcription, the transcription products from five eukaryotic expression plasmids (Fig. 7 A (I) and 7 (II)) were evaluated by real-time PCR (Fig. 7 B, C, D, E, F & G), results from which was in accordance with the data presented in figure 6 A, suggesting that the suppressive effect of BBR on gene transcription might be gene-nonspecific.

### Berberine can inhibit the transcription of DNA in a cell-free system

**Figure 8:**
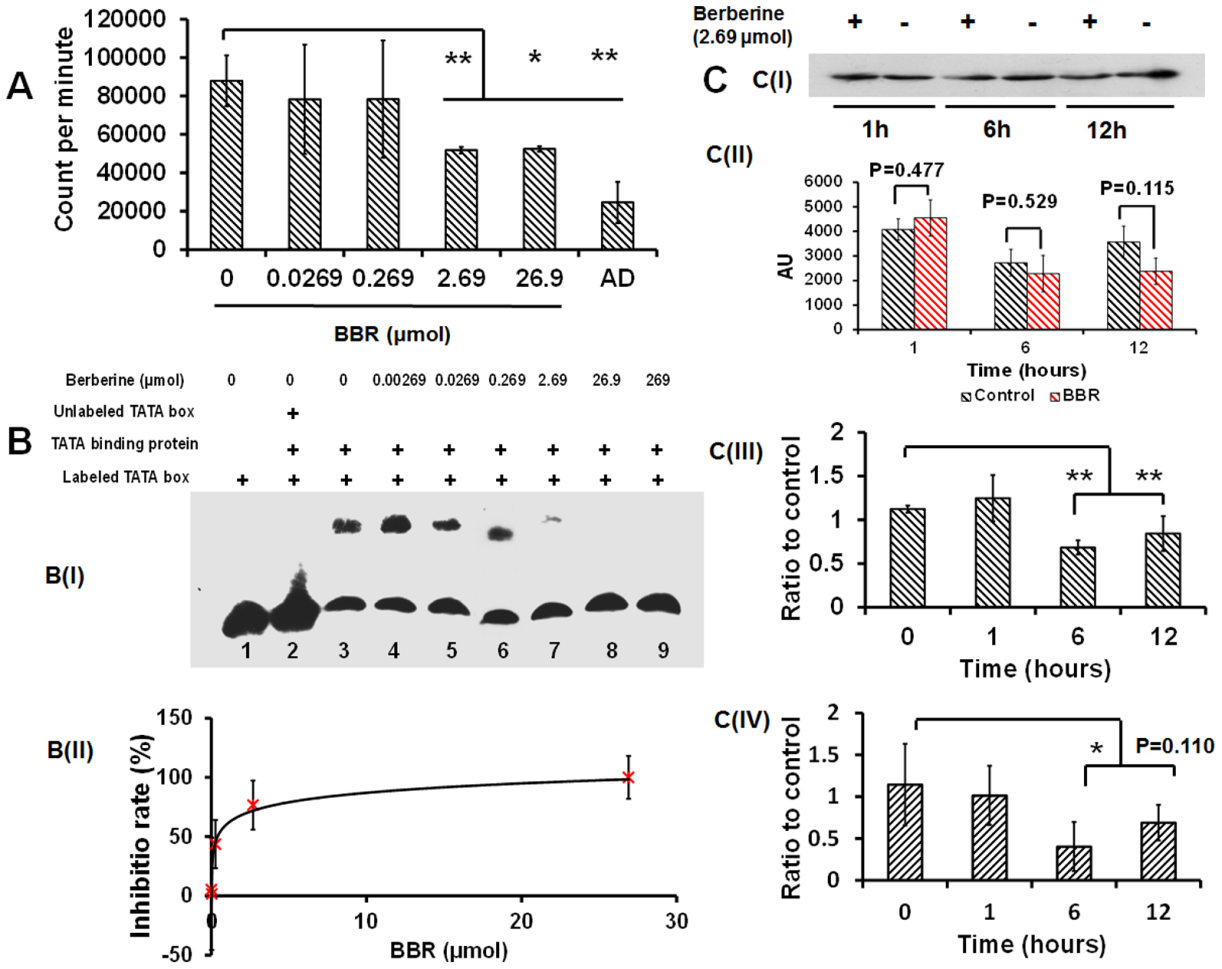
The association of BBR with DNA can suppress the interaction between TBP and the TATA box. (A) represents the suppressive effect of BBR on gene transcription in a cell-free system, where actinomycin D (AD) was used as the positive control drug because of its well-known transcription inhibitory activity. (B) represents the suppressive effect of BBR on the association between TBP and the TATA box. B (I) is an image taken via EMSA, and B (II) represents the effect of BBR on the binding of TBP to TATA box, (y = 11.468×, ln-(x)*+*.*60.55*, R^2^ = 0.9344, n = 3. (C) represents the time-dependent suppressive effect of BBR (2.69 µmol) on the interaction between TBP and the TATA box in live cells observed using ChIP. C (I) is one of the images taken from a Western blot representing TBP, and C (II) displays the statistical results of brightness of TBP in Western blot images from three independent experiments. C (III) and C (IV) depict the quantitative analysis of the TATA box-containing DNA fragment in the CMV promoter and PPARγ promoter bound by TBP from four independent experiments, the data of which was represented as the ratio of the content of DNA fragments in BBR groups to the content in BBR-free groups at the same time-point (control group), input of each sample was used as inner reference. * P<0.05, ** p<0.01 vs the corresponding control indicated above. Data were presented as mean ± S.D, from three independent experiments (n = 3).

A living cell is a complex system composed of numerous known and unknown bioactive components. To reject the possibility that components in the cytoplasm facilitate the process of BBR suppressing gene transcription, a cell-free transcription system was used. In accordance with results stated previously in the living cell system, a dose-dependent suppression of DNA-template transcription was observed (Fig. 8 A): BBR can significantly inhibit the transcription of the DNA template with dosages of 2.69 µmol and 26.9 µmol, even when devoid of a cytoplasm as in a Hela cell.

### Berberine can inhibit TATA binding protein from binding to the TATA box

To validate the suppressive effect on gene transcription and to determine BBR's target site during interaction with DNA, EMSA was employed by using oligonucleotides containing the TATA box core sequence as a probe. Results from this study demonstrated that BBR's association with the TATA box-containing oligonucleotides could dramatically reduce the binding affinity of TATA binding protein (TBP) to the TATA box at a dosage of 0.269 µmol (Fig. 8 B (I)), and the IC50 value was 0.3985 µmol (Fig. 8 B (II)). Furthermore, the suppressive effect of BBR on TBP binding to the TATA box of the CMV promoter, without fluctuation in TBP content (Fig. 8 C (I) & C (II)), was also observed in living cells 6 hours after addition of BBR (2.69 µmol) to the growth medium by means of a ChIP assay (Fig. 8 C (III) & C (IV)), indicating that the association with DNA is the central reason for the suppression of gene transcription observed in this entire study.

## Discussion

Previous literature coupled with cytotoxicity data obtained from our laboratory have revealed that at a concentration of 2.69 µmol, BBR is within a safe dosage range for PC12 cells and is also the minimum effective dosage for protecting cultured neuron-like PC12 cells from damage after oxygen and glucose deprivation [4]. In order to investigate the effects of BBR on DNA transcription with more relevance to its numerous reported pharmacological functions, the dosage of 2.69 µmol was used according to previous experiments in living cells.

According to the images obtained from the confocal microscope, the absorption of BBR by PC12 cells from the medium is a very rapid process, which can be observed 30 minutes after drug administration by measuring BBR's fluorescence intensity in the nucleus. This is the first direct observation that BBR can enter a living cell and distribute throughout the cytoplasm as well as the nucleus. This evidence for BBR's existence within the cell provides solid support for our hypothesis that BBR's suppressive effect of gene transcription is based on its association with DNA. This result was the basis for our choice of 30 minutes as the first time-point to study the effect of BBR on gene expression. The curve representing the time-dependent concentration of berberine in PC12 cells was similar to the one reported by our lab previously in primary cultured cerebral neurons [11].

Quantitative analysis of the global RNA levels in different trials revealed that BBR can effectively suppress the transcription level in living cells on a global scale within 30 minutes after BBR-treatment, which is in accordance with the time needed for BBR to enter the nucleus. Coupled with the discovery in this study that BBR can swiftly alter the spatial conformation of testing components (chromatin, plasmids, genomic DNA) after associating with them, the inhibitory effect of BBR on DNA transcription was inferred, and the interaction between BBR and DNA is considered the direct cause for this suppressive activity of BBR.

To acquire a more thorough understanding regarding the effect of BBR on gene transcription, five different promoter-driven expression plasmids (Fig. 7 A (I) & A (II)) were used as models for the following purposes: two different fluorescent protein expressing plasmids (CMV-GFP and CMV-RFP) driven by the CMV promoter (TATA box-dependent) were used to reject the possibility that BBR has coding sequence specificity; an IgG promoter (TATA box-independent) and a PPARγ promoter (TATA box-dependent) driving green fluorescent protein expressing plasmids were used to reject the possibility that BBR has promoter sequence specificity; and a TPH2 promoter (TATA box-dependent) driving red fluorescent protein expressing plasmid was used to add a random sample to support our conclusion. Considering the potential suppressive effect of BBR on gene transcription, the mRNA levels of a target gene were presented as the ratio of the target gene in the BBR group to the same target gene in the BBR-free group at the same time-point. According to this relative quantitative analysis, the result of BBR suppressing the transcription of all five plasmids suggests the conclusion that BBR exhibits a suppressive effect on gene transcription in a non-specific fashion. However, an interesting question was also raised at the same time: Despite the time-point at 1 hour after drug administration, we may inquire why BBR's suppressive effect on transcription was not universally observed in all plasmids at subsequent time-points, which differs from the observed suppressive effect of BBR on global RNA synthesis? This phenomenon revealed to us that BBR could effectively suppress the expression of gene in a short-term fashion but this level of activity varies in the long-term. This variable effect of BBR on different promoters after long-term incubation is related to the intricate and complex interactions between many other regulatory mechanisms that may counter the suppressive effect of BBR by up-regulating gene expression, such as the protective effect of BBR on mRNA from degradation, causing the suppressive effect to be neutralized to different intensities by such a counteracting mechanism. We believe that the variances in the long-term effect of BBR on these candidate promoters represent the adaptability and responses of a living system to an extraneous stimulus. Although an unresolved problem remains to be settled here, the non-specific suppressive effect of BBR on gene transcription, whether containing the TATA box sequence or not, can still be confirmed.

With the elimination of BBR-treatment, the suppressive effect of BBR on gene transcription was removed in a time-dependent fashion (Fig. 6 A (II)), and the slopes indicating the increase in RNA level between the BBR group and control group were similar to each other. A cytometric analysis also demonstrated that the suppressive effect of BBR on gene transcription is reversible (Fig. 5 C), suggesting that the inhibitory effect of BBR on gene expression is due to the suppression of transcription, rather than the complete eradication of transcriptional ability. There are two possible explanations for the cessation of the suppressive effect on gene transcription after the removal of BBR-treatment: 1) the export of BBR out of cells by P-glycoprotein [12], and 2) the weak, non-covalent interactions between BBR and double-stranded DNA reported previously [24].

The in vitro transcription system used in this study is the nuclear extract of a Hela cell without cytoplasm. The DNA template used in this experiment is an artificial gene driven by a CMV promoter, which contained the TATA box sequence and was identical to the promoter driving GFP and RFP expression in living cells. Although the involvement of cytoplasmic factors alongside BBR in the suppression of gene transcription in living cells cannot be rejected by this system, data from this study illustrates that the distribution of BBR in the nucleus can suppress gene transcription without the presence of the cytoplasm.

The TATA box is a *cis*-regulatory element involved in the process of gene transcription and can associate with TBP, allowing the RNA polymerase II to unwind the DNA while recruiting other transcriptional factors and subunits to form the transcriptional complex to ultimately initiate transcription [25]. Due to the disruptive effect of berberine on the interaction between TBP and the TATA box, which was demonstrated by EMSA in vitro and ChIP in living cells, there are at least two facts that can be concluded: 1) the interaction between TBP and the TATA box is one target of BBR in living cells, and 2) due of the fact that more than 23.85% of eukaryotic promoters contain the TATA box sequence [26], the suppressive effect of BBR on gene transcription has no single-gene-specificity. Considering the architecture of the minor groove of DNA for TBP binding to the TATA box [27], the competitive obstruction of the TBP binding site by the non-covalent, stereospecific insertion of BBR into the minor groove of DNA is inferred as the mechanism for the suppressive effect of BBR on gene transcription. Furthermore, because of the substantial possibility that the spatial conformational change of DNA induced by a DNA intercalator may affect DNA-protein interactions [28], the BBR-induced spatial conformational change of DNA may also be a basis for the obstruction of binding between TBP and the TATA box.

Taking everything together, this is the first report to describe a general and universal effect of BBR on living biological systems in detail: from the specific chemical features to the biological functional level. The detection of the DNA binding character with BBR and the suppressive effect on the association between TBP and TATA box by BBR suggests that the non-specific suppressive effect on gene transcription by BBR is a direct result of its chemical features. Because the concentration of BBR in a living cell's nucleus could exceed 2.69 µmol at 2 hours following drug administration, the acquired results from the living cell system and cell-free system can complement each other and support the integrity of the entire study. Due to the definite association between BBR and TATA box in living cells suffering from BBR treatment, we believe that the non-specific suppressive effect on gene transcription is one of the fundamental mechanisms employed in the numerous pharmacological functions of BBR that are exhibited in living systems. For instance, the suppression of the PPARγ promoter by BBR, which was discovered in this study, is in good agreement with a previous report [29] describing the possible mechanism of BBR inhibiting 3T3-L1 adipocyte differentiation.

In contrast to our hypothesis, however, not all genes are down-regulated after cell suffering from BBR treatment in previous studies, including *pgp-170* in human hepatoma cell lines [30], *gata-3* and *gata-2* in 3T3L1 cell lines [31], *insR* in type 2 diabetes animal models [32], *wee1* in leukemia cells [33], and *ldlr* in hepatic cells [34], etc. All of the promoters in these genes lack the canonical TATA box sequence. However, coupled with the suppressive effect of BBR on the TATA box-independent IgG promoter that was discovered in this study, the possibility that BBR suppresses TATA box-independent genes cannot be excluded because no evidence has been made available to demonstrate that these TATA box-independent genes are up-regulated through their direct interaction with BBR. Therefore, the current theory concerning BBR's effect on TATA box-independent genes still remains to be settled. Moreover, the mechanism involved in the various drug responses by different genes is an interesting field that requires much further work to completely study and understand. Lastly, we would like to inquire what the role of such a simple and definite function might be in generating an array of complex pharmacological activities in a biological system. This would be due to the complicated functional and structural features of the biological system. We believe that this work can shed new light on the pharmacological mechanisms of chemicals within living cells, such as berberine, regarding the implementation of numerous activities based on few specific targets.

## References

[1] Kuo CL, Chi CW, Liu TY (2004). The anti-inflammatory potential of berberine in vitro and in vivo.. Cancer Lett.

[2] Xu LH, Liu L, He XH (2005). Inhibitory effects of berberine on the activation and cell cycle progression of human peripheral lymphocytes.. Cellular and molecular immunology.

[3] Yin J, Xing HL, Ye JP (2008). Efficacy of berberine in patients with type 2 diabetes mellitus.. Metabolism Clinical and Experimental.

[4] Zhou XQ, Zeng XN, Kong H, Sun XL (2008). Neuroprotective effects of berberine on stroke models in vitro and in vivo.. Neurosci Lett.

[5] Bova S, Padrini R, Goldman WF, Berman DM, Cargnelli G (1992). On the mechanism of vasodilating action of berberine: possible roleof inositol lipid signaling system.. J Pharmacol Exp Ther.

[6] Chiou WF, Yen MH, Chen CF (1991). Mechanism of vasodilatory effect of berberine in rat mesenteric artery.. Eur J Pharmacol.

[7] Kuo CL, Chou CC, Benjamin YM (1995). Berberine complexes with DNA in the berberine-induced apoptosis in human leukemic HL-60 cells.. Cancer Lett.

[8] Krey AK, Hahn FE (1969). Berberine: complex with DNA.. Scinece.

[9] Stockert JC (1985). Cytological effects of berberine sulphate on chironomus salivary gland nuclei.. Chromosoma.

[10] Bhadra K, Maiti M, Kumar GS (2008). Berberine-DNA complexation: new insights into the cooperative binding and energetic aspects.. Biochimica et biophysica acta.

[11] Wang XL, Xing DM, Wang W, Lei F, Su H (2005). The uptake and transport behavior of berberine in Coptidis Rhizoma extract through rat primary cultured cortical neurons.. Neuroscience Letters.

[12] Chen YY, Wang XL, Sun H, Xing DM, Hu J (2008). Characterization of the transportation of berberine in Coptidisrhizoma extract through rat primary cultured cortical neurons.. Biomed Chromatogr.

[13] Blobel G, Potter VR (1966). Nuclei from rat liver: isolation method thatcombines purity and high yield.. Science.

[14] Bonner J, Chalkley GR, Dahmus M, Fambrough D, Fujimura F (1968). Isolation and characterization of chromosomal nucleoproteins.. Method Enzymol.

[15] Mosmann T (1983). Rapid colorimetric assay for cellular growth and survival: application to proliferation and cytotoxicity assays.. J Immunol Methods.

[16] Wang YG, Lei F, Wang XK, Hu J, Zhan HL (2009). Regulatory effects of Wuzhuyutang (Evodiae prescription) and its consisting herbs on TPH2 promoter.. China Journal of Chinese Materia Medica.

[17] Wang YG, Li LL, Lei F, Liang AH, Xing DM (2010). Study on IgG promoter as probe to evaluate safety of injections in pre-clinic.. China Journal of Chinese Materia Medica.

[18] Kheir MM, Wang YG, Hua L, Hu J, Li LL (2010). Acute toxicity of berberine and its correlation with the blood concentration in mice.. Food Chem Toxicol.

[19] Akihiko T, Yoshihiro S, Masahiko H, Takayuki S, Hiroyuki K (2001). Development of a time-resolved fuloreometric method for observing hybridization in living cells using fluorescence resonance energy transfer.. Biophys J.

[20] Chiang SY, Welch J, Rauscher FJ, Beerman TA (1994). Effects of minor groove binding drugs on the interaction of TATA box binding protein and TFIIA with DNA.. Biochemistry.

[21] Li HY, Hu J, Ma L, Yuan ZY, Wang YG (2010). Comprehensive study of baicalin down-regulating NOD2 receptor expression of neurons with oxygen-glucose deprivation in vitro and cerebral ischemia-reperfusion in vivo.. Eur J Pharmacol.

[22] Mazzini S, Bellucci MC, Mondelli R (2003). Mode of binding of the cytotoxic alkaloid berberine with the double helix oligonucleotide D(AAGAATTCTT)_2_.. Biooranic and medicinal chemistry.

[23] Li WY, Liu ZH (1998). The fluorescent reaction between berberine and DNA and the fluorometry of DNA.. Microchem J.

[24] Huang C, Zhang YB, Gong ZW, Sheng XY, Li ZM (2006). Berberine inhibits 3T3-L1 adipocyte differentiation through the PPARγ pathway..

[25] Stephen KB (1996). The TATA box binding protein.. Curr Opin Struc Biol.

[26] Zhang XH, Qi YX (2008). Analysis on TATA-box, GC-box and CAAT-box in eukaryotic promoters..

[27] Patikoglou GA, Kim JL, Sun LP, Wang SH, Kodadek T (1999). TATA element recognition by the TATA box-binding protein has been conserved throughout evolution.. Gens & Development.

[28] Stephen N (2001). DNA minor-groove recognition by small molecules.. The royal society of chemistry.

[29] Chen WH, Qin Y, Cai Z, Chan CL, Luo GA (2005). Spectrometric studies of cytotoxic protoberberine alkaloids binding to double-stranded DNA.. Bioorganic and medicinal chemistry.

[30] Lin HL, Liu TY, Liu WY, Chi CW (1999). Up-regulation of multidrug resistance transporter expression by berberine in human and murine hepatoma cells.. Cancer.

[31] Hu YS, Davies GE (2010). Berberine inhibits adipogenesis in high-fat diet-induced obesity mice.. Fitoterapia.

[32] Zhang H, Wei J, Xue R, Wu JD, Zhao W (2010). Berberine lowers blood glucose in type 2 diabetes mellitus patients through increasing insulin receptor expression.. Metabolism.

[33] Lin CC, Lin SY, Chung JG, Lin JP, Chen GW (2006). Down-regulation of cyclin B1 and up-regulation of Wee1 by berberine promotes entry of leukemia cells into the G2/M-phase of the cell cycle.. Anticancer Res.

[34] Lee S, Lim HJ, Park JH, Lee KS, Jang Y (2007). Berberine-induced LDLR up-regulation involves JNK pathway.. Biochem Bioph Res Co.

